# Dengue virus IgG and serotype-specific neutralizing antibody titers measured with standard and mature viruses are associated with protection

**DOI:** 10.21203/rs.3.rs-4145863/v1

**Published:** 2024-04-12

**Authors:** Leah Katzelnick, Camila Odio, Jedas Daag, Maria Vinna Crisostomo, Charlie Voirin, Ana Coello Escoto, Cameron Adams, Lindsay Dahora Hein, Rosemary Aogo, Patrick Mpingabo, Guillermo Raimundi Rodriguez, Saba Firdous, Maria Abad Fernandez, Laura White, Kristal-An Agrupis, Jacqueline Deen, Aravinda de Silva, Michelle Ylade

**Affiliations:** Viral Epidemiology and Immunity Unit, Laboratory of Infectious Diseases, National Institute of Allergy and Infectious Diseases, National Institutes of Health; Viral Epidemiology and Immunity Unit, Laboratory of Infectious Diseases, National Institute of Allergy and Infectious Diseases, National Institutes of Health; Institute of Child Health and Human Development, National Institutes of Health, University of the Philippines Manila, Manila, Philippines; Institute of Child Health and Human Development, National Institutes of Health, University of the Philippines Manila, Manila, Philippines; Viral Epidemiology and Immunity Unit, Laboratory of Infectious Diseases, National Institute of Allergy and Infectious Diseases, National Institutes of Health; Viral Epidemiology and Immunity Unit, Laboratory of Infectious Diseases, National Institute of Allergy and Infectious Diseases, National Institutes of Health; University of North Carolina at Chapel Hill; Department of Microbiology and Immunology, University of North Carolina School of Medicine, Chapel Hill; Viral Epidemiology and Immunity Unit, Laboratory of Infectious Diseases, National Institute of Allergy and Infectious Diseases, National Institutes of Health; Viral Epidemiology and Immunity Unit, Laboratory of Infectious Diseases, National Institute of Allergy and Infectious Diseases, National Institutes of Health; Viral Epidemiology and Immunity Unit, Laboratory of Infectious Diseases, National Institute of Allergy and Infectious Diseases, National Institutes of Health; National Institute of Allergy and Infectious Diseases; Department of Microbiology and Immunology, University of North Carolina School of Medicine, Chapel Hill; Department of Microbiology and Immunology, University of North Carolina School of Medicine, Chapel Hill; Institute of Child Health and Human Development, National Institutes of Health, University of the Philippines Manila; Institute of Child Health and Human Development, National Institutes of Health, University of the Philippines Manila; University of North Carolina at Chapel Hill; Institute of Child Health and Human Development, National Institutes of Health, University of the Philippines Manila

## Abstract

Recent work demonstrates the limitations of the standard dengue virus (DENV) neutralization assay to predict protection against dengue. We perform studies to compare how a commercial IgG ELISA, envelope domain III (EDIII) or non-structural protein 1 (NS1) binding antibodies, and titers from plaque reduction neutralization tests (PRNTs) using reference standard and clinical mature viruses are associated with dengue disease. Healthy children (n = 1,206) in Cebu, Philippines were followed for 5 years. High ELISA values (≥3) were associated with reduced dengue probability relative to naïve children (3% vs. 10%, p = 0.008), but antibody binding EDIII or NS1 from each serotype had no association. High standard and mature geometric mean PRNT titers were associated with reduced dengue disease overall (p < 0.01), and high DENV2 and DENV3 titers in both assays provided protection against the matched serotype (p < 0.02). However, while 52% of dengue cases had standard virus PRNT titers > 100, only 2% of cases had mature virus PRNT titers > 100 (p < 0.001), indicating a lower, more consistent threshold for protection. Each assay may be useful for different purposes as correlates of protection in population and vaccine trials.

## Introduction

Dengue is a febrile illness caused by any one of four co-circulating viruses, dengue virus serotypes 1–4 (DENV1–4), with disease ranging from undifferentiated fever to severe manifestations including vascular leakage, shock, bleeding, and organ impairment^1^. An estimated 75% of infections are asymptomatic^2^, and these inapparent infections likely contribute to viral spread. DENV1–4 are transmitted among humans by mosquitos in > 100 tropical and subtropical countries. Globally, at least 2.5 billion people are at risk for dengue, and dengue is one of few infectious diseases with a rising incidence in this century^3, 4^. Between 1990 and 2019, the global number of dengue cases increased by 85% to 56.88 million in 2019^5^. The heavy healthcare burden and rising incidence have motivated dengue vaccine development for decades, but these have provided incomplete protection, especially among individuals with no DENV antibodies at the time of vaccination^6, 7^.

An important limiting factor in the development of dengue vaccines is the lack of widely accepted correlates of protection. In the context of vaccines, correlates of protection are biomarkers that can be induced by immunization and reliably predict vaccine efficacy in preventing a clinical outcome^8, 9^.Because correlates of protection are often easier and faster to measure than the desired clinical outcome, they can expedite trial results and vaccine approvals^10^. Once identified, regulators prefer that a correlate of protection have a single threshold that distinguishes those who get disease from those who do not get disease or even infection, but this can be complicated by heterogeneity in exposures and responses^11^.Additionally, although an intervention meets the correlate of protection threshold at one time point, this may not persist due to waning immune responses. Thus, although a goal biomarker level is intuitive, a relative correlate of protection, where the probability of disease decreases gradually with increasing levels of the immune marker, may be more realistic^11^.To establish a correlate of protection, a biological assay is often first identified as a correlate of risk in longitudinal cohort studies. Correlates of risk are biomarkers associated with both natural infection and disease outcomes, and it is important to distinguish between markers of immune protection versus those that simply predict the probability of exposure to the pathogen. Controlling for covariates that independently explain disease risk (e.g. age, sex, measures of local transmission intensity, etc.) help build evidence that a correlate of risk could be a plausible correlate of protection^9^.

For dengue vaccines, correlates of protection have not been widely accepted due to the inability to identify a single threshold, the complexities of the four interactive and immunomodulatory serotypes, and fundamental differences in vaccine responses between dengue seronegative and seropositive individuals. Numerous studies by Gilbert and colleagues have established that neutralizing antibody (nAb) titers measured using standard plaque reduction neutralization tests (PRNT) are correlates of protection in phase 3 dengue vaccine efficacy trials^12, 13, 14^. However, a single nAb threshold cannot be identified because even some individuals with exceptionally high titers (e.g. >500) remain at risk of disease^14^. This contrasts with other vaccines against flaviviruses. For instance, a PRNT titer of 1:5 is protective against YFV infection and 1:10 against JEV infection, while 125 ELISA units is protective against TBEV infection^11^. Moreover, since DENV nAbs must be measured against all four serotypes, it is unclear whether correlates are different for each serotype or if a common correlate is feasible. Finally, correlates of protection against dengue may not be binary due to antibody-dependent enhancement (ADE) where low to intermediate levels of antibodies can increase risk of severe disease compared to those with no antibodies or high antibodies.

Recent work indicates that antibody quality may be playing a critical yet underexplored role in dengue correlates. The validated (standard) PRNTs for measuring correlates of protection in dengue vaccine trials cannot distinguish between high quality antibodies that are mechanistically protective and lower quality antibodies that may increase risk of severe disease. There is emerging consensus that type-specific, or homotypic, nAbs induced by vaccines provide protective efficacy against dengue, and this population can be profiled using antibody depletion assays^15, 16^. Alternatively, cross-reactive antibodies target multiple serotypes and can either be low quality and associated with enhancement or high quality and broadly neutralizing^17, 18^. Assays that use mature rather than standard viruses to measure nAbs may better distinguish antibody quality and serve as more accurate correlates of protection^19^.

Specifically, dengue virions are assembled as immature virions containing 60 copies of envelope (E) and pre-membrane (prM) heterotrimeric spikes on the surface. During virus egress from cells, prM is cleaved to generate an infectious mature particle containing 90 E homodimers that lie flat on the viral surface. The cleavage of prM is inefficient in laboratory cell lines and the standard virions used in the PRNT assay are heterogenous and display prM and fusion loop epitopes. Standard virions are sensitive to neutralization and/or enhancement by antibodies to fusion loop or prM epitopes, which are hidden or absent in fully mature virions^17, 20^. While the processing and maturation state of DENV in humans remains to be well defined, a recent study has demonstrated that DENV1 in humans is fully mature and insensitive to fusion loop and prM antibodies^21^. Mature virions lack prM, resulting in only intermittent exposure of the fusion loop and a dimer configuration of the E protein that binds potent, type-specific antibodies. Compared to standard virions, an assay using mature virions is likely to be more selective, measure potent nAbs, and be less sensitive to neutralization or enhancement by fusion loop and prM antibodies^18^.

Here, we evaluate multiple antibody measures as correlates of risk in the context of a natural infection cohort study in the Philippines. We examine a commercial IgG ELISA (PanBio; Brisbane, QLD), binding antibodies to E domain III (EDIII) or non-structural protein 1 (NS1) from each serotype, a PRNT assay using the reference strains recommended by the World Health Organization (WHO) and grown in standard laboratory conditions (standard), and a PRNT assay using circulating Southeast Asian strains (clinical) grown in conditions that induce mature virions as a more plausible ‘mechanistic’ correlates of risk. Our work compares multiple antibody markers as potential correlates of risk for use in future vaccine trials and for measuring protection and risk in natural dengue settings.

## Methods

### Ethics statement

The study protocol was approved by the University of the Philippines – Manila Research Ethics Board and is registered at clinicaltrials.gov (NCT03465254). Written informed consent was obtained from a parent or legal guardian, and verbal assent was obtained from participants.

### Participants and baseline characteristics

Children aged 9–14 years and residing in Bogo or Balamban, Cebu, Philippines were recruited for a longitudinal observational study examining dengue risk among those who were eligible to receive CYD-TDV (Dengvaxia, Sanofi Pasteur)^22^. Serum samples were collected from 2,996 enrolled participants between May 2 and June 2, 2017, and febrile surveillance occurred between November 1, 2017 and October 31, 2022. All participants had basic demographic information collected, and febrile cases were evaluated with a case report form and reverse transcriptase polymerase chain reaction (RT-PCR) for DENV1–4^23^. To assess the impact of natural immunity on the probability of dengue, only unvaccinated children were included in these analyses (n = 1,206). There were 57 cases of symptomatic dengue among the unvaccinated (4.7%). Seven participants had a second dengue case during the observation period, but only the first case was considered here. Cases were classified by the 2009 WHO dengue criteria as Dengue without Warning Signs (DwoWS), Dengue with Warning Signs (DWWS), and Severe Dengue. All baseline participant data were summarized using the gtsummary package. Means were compared using two sample t-test, and proportions were compared using Pearson’s Chi-squared test.

### Sample selection

We designed a series of case-control studies to evaluate each serological assay as a correlate of dengue risk. We tested all available samples from individuals who progressed to symptomatic dengue in any immune group. Individuals who did not experience dengue during the study were included as controls. Given that many of these assays are labor intensive, subsets of the controls were tested by each immune assay, and these were chosen in the following manner. Fifirst, baseline dengue immunity was evaluated in all individuals by indirect DENV IgG enzyme-linked immunosorbent assay (ELISA, PanBio; Brisbane, QLD, Australia). Since this ELISA uses inactivated DENV1–4 viruses as the antigen, it detects total antibodies^24^. Next, testing by standard reference PRNT was performed on random selection of 34% of participants with ELISA values < 0.2, 100% with ELISA 0.2–3, and 30% with ELISA > 3 (Supplemental Table 1). Samples that neutralized at least 70% of infection at a serum dilution of 1:40 were considered immune to the tested serotype. Neutralization of one serotype was considered monotypic while neutralization of > 1 serotype was labeled multitypic immunity. Samples that did not neutralize any serotype were considered naïve^22^.

Subsets from these naïve, monotypic, and multitypic groups were then randomly selected for the assessment of nAb by mature PRNT, binding NS1 and EDIII antibodies, and the occurrence of inapparent infections (Supplemental Table 2). To account for the different proportions of samples tested within each baseline immune status group, we used inverse probability weighting in all models to ensure the sample was representative of the cohort. Additionally, to have a consistent comparison group across models, we assumed undetectable mature PRNT and NS1 and EDIII antibodies in all individuals with ELISA < 0.2 (n = 98) or undetectable antibodies by standard PRNT (n = 40). Validation testing supported this assumption. Specifically, among individuals with ELISA < 0.2, 99% were naïve by standard PRNT (n = 78) and 100% were naïve by mature PRNT (n = 36).

### Standard and mature PRNT assays

To facilitate the analysis of many samples, the standard PRNT was simplified to consist of only two dilutions (1:40 and 1:200, hereafter reported as 40 and 200)^22^. Antibody titers were measured using DENV1–4 WHO reference strains (DENV1 WP74, DENV2 S16803, DENV3 CH53489, DENV4 TVP-376) grown in C6/36 cells, and this assay is referred to as the standard reference PRNT (standard, where not otherwise indicated). PRNT_50_ titers were estimated from the two-dilution assay in comparison to control wells with virus but no serum using the Reed-Muench method^25^.

To assess nAbs against mature virions, we used low-passage DENV strains that matched the genotypes circulating in the Philippines during the study period (GenBank accession numbers AIE17470.1, QBP33521.1, AFI55000, and AHN50410). We generated mature virus by propagating these viruses in Vero-furin cell lines as previously described, and this assay is referred to as clinical mature (mature)^26, 27^. The PRNT was performed as described previously^28^. Briefly, 1.7×10^4^ Vero cells per well were plated and incubated at 37°C overnight. Human immune sera were fourfold serially diluted over a range from 1:20 to 20480 and mixed with Vero-furin-produced low passage clinical isolates of DENV1–4. Sera-virus mixtures were incubated at 37°C for one hour, then added to confluent cells for a one-hour inoculation. Subsequently, 1% methycellulose overlay was added, and cells were incubated for 2 days. Cells were fixed in 80% methanol, blocked in 5% non-fat dried milk, and immunostained using mouse 4G2 and 2H2 antipan in avivirus monoclonal antibodies, secondary horseradish peroxidase (HRP)-labelled goat anti-mouse IgG antibody (Jackson ImmunoResearch Laboratories), and developed using TrueBlue HRP substrate. Images of all wells were collected using the Cellular Technology Limited (CTL) machine and ImmunoSpot software and automated plaque counting was performed using the Viridot plaque counter package in R^29^. PRNT_50_ titers were estimated by 4-parameter logistic regression at 50% reduction in plaque count relative to virus only control wells using the drc package in R.

For both the standard and mature PRNT, those with PRNT_50_ titers outside the assay range were set as a two-fold dilution below or a two-fold dilution above the assay limit of detection. Geometric mean titers (GMT) were calculated as the average of the log transformed DENV1–4 nAb titers. To validate the two-dilution standard nAb titers, we reduced the full-dilution data from the mature PRNT to only 40 and 200 dilutions and estimated PRNT_50_ titers using the Reed-Muench method. PRNT data from full dilution and two-dilution methods were highly correlated (Supplemental Fig. 1). Pearson correlations between full dilution and two-dilution mature serotype-specific titers and GMT were assessed using the ggpubr package and plotted with ggplot2. Only samples within the limit of detection of the assay were included in these comparisons. This analysis was performed on multitypic individuals only since these are the most immunologically complex sera.

The frequency of inapparent infection was assessed in a subset of participants (48 naïve, 42 monotypic, and 66 multitypic individuals) by comparing mature PRNT_50_ titers between samples collected at baseline versus those collected approximately 1 year after enrollment (Supplemental Table 2). Seroconversion or a 4-fold increase in mature PRNT_50_ against any serotype without documented clinical symptoms was labeled an inapparent infection^30^.

To assess the impact of strain and maturation state on nAb titers, a direct comparison of nAb titers against mature and standard WHO reference and circulating clinical strains was performed. Each reference and clinical strain was grown in Vero cells to produce standard isolates with prM proteins present and in furin-overexpressing Vero cells to produce fully mature viral particles. Six-dilution PRNT assays were performed as described above on a random subset of individuals with baseline multitypic (n = 30) or monotypic immunity (n = 6). The resulting GMT and serotype-specific titers against each standard and mature WHO and clinical strain were plotted using ggplot2 and ggpubr packages and compared using pairwise paired t-tests with a Bonferroni correction for multiple comparisons.

### Luminex multiplex assays

Antibody responses to envelope protein domain 3 (EDIII) and nonstructural 1 protein (NS1) of DENV serotypes 1–4 were measured in a random subset of participants (n = 295, Supplemental Table 2) using Luminex multiplex assay as previously described^31^. Briefly, biotinylated EDIII antigens and biotinylated bovine serum albumin (BSA) were coupled to unique MagPlex^®^-Avidin Microspheres (Luminex) while His-tagged NS1 antigens (The Native Antigen Company) were coupled following immobilization of anti-His tag antibody (abcam) onto unique avidin-coated microspheres. The panel of EDIII, NS1 and BSA conjugated microspheres were mixed in equal ratios and plated at 2,500 beads per antigen in 50μL/well in 96 well plates. Diluted human serum (1:500) was incubated with antigen-conjugated microspheres for one hour at 37°C, 700rpm. Later, immune complexes were incubated with goat anti-human IgG Fc multispecies SP ads-PE antibody (Southern Biotech) following three washes. Antibody responses were detected using a Luminex 200 analyzer and expressed as median fluorescence intensity after subtracting the non-specific antibody binding signal (to BSA). Selected samples from healthy donors and well-characterized DENV and ZIKV seropositive individuals were run on multiple assay plates to verify assay performance and assess inter-assay variability.

### Correlates of risk analyses

All analyses were performed with RStudio for macOS (2022.07.1, Build 554). To account for potential non-linear relationships between antibody titers and outcomes, we estimated the probability of symptomatic dengue, dengue without warning signs, and dengue with warning signs as functions of baseline ELISA IgG and the geometric mean of standard and mature PRNT titers on both discrete and continuous scales using logistic regression in the Poisson distribution. Inverse probability weighting was used to adjust for the number of sampled individuals within each immune group. Continuous relationships were modeled using the mgcv package to create generalized additive models (GAMs) with 95% confidence intervals (CI) to allow for non-linear effects. The logistic regression models for binned values were generated using the stats package. Antibody bins for ELISA data were chosen considering the manufacturer recommended cut-point of > 1.1 as DENV seropositive and equalizing the numbers of samples in each bin as much as possible (Supplemental Table 3). For standard GMT data, antibody bins were chosen based on the two dilution points of 40 and 200 and an intermediate point of 100, which was noted by Gilbert et al. as the titer where dengue vaccine efficacy (Dengvaxia, Sanofi Pasteur) was consistently above 50%^14^. For mature GMT data, antibody bins were chosen based on the lowest dilution point of 20 and the intermediate point of 100. The probabilities from the GAMs and binned logistic regression models were then combined and graphed using the ggplot2 package. The predicted disease probabilities from each model were obtained using the binomial distribution and ggeffects package.

Because inapparent infections and DENV2 and DENV3 cases were less frequent events, the odds of each outcome was assessed by baseline standard and mature PRNT and serotype-specific EDIII and NS1 binding antibodies as continuous, linear predictors using the stats package and inverse probability weighting. Forest plots were generated using ggplot2. All models used the Poisson distribution and were adjusted for age, sex, and enrollment site. Dengue probabilities are shown for the average study participant (female, age 10, from Bogo). All models were assessed for collinearity among predictors by using the car package to calculate the variance inflation factors, which were < 5 indicating no significant collinearity.

To evaluate how models may have been affected by inverse probability weighting, we limited the ELISA dataset to values only from those with binding antibody (Luminex, n = 295), standard (n = 823) or mature (n = 293) nAb titer data. We then built models of each of these datasets adjusted by inverse probability weighting and bootstrapping, and compared the results to those gained from the model of the full ELISA dataset. Bootstrapping was performed using the boot package in R wherein subsets were resampled 2,000 times and CI were calculated using bias-corrected and accelerated bootstraps. CI from the full dataset were largely overlapping with the models from weighted and bootstrapped datasets (Supplemental Fig. 2). Thus, the effect sizes from the models using weighted binding and nAb as predictors likely reflect the true effect sizes from the full dataset.

## Results

### Baseline characteristics

We first evaluated whether baseline demographic characteristics and immune status were associated with risk of symptomatic dengue in this population. There were no differences in age at enrollment, gender, or residential site between those that developed symptomatic dengue (including dengue without warning signs, DwoWS, and dengue with warning signs, DWWS) and those that did not (Table 1). Although most of the participants had multitypic immunity by standard PRNT, the dengue attack rate was highest in the monotypic group (9.6%), followed by the naïve (7.2%) and multitypic groups (3.7%) (p = 0.005). Those who developed symptomatic dengue had lower IgG ELISA values (p = 0.036). Of the 57 cases, there was no severe dengue, 32 had DWWS (DENV1, n = 8/12 cases; DENV2, n = 9/17; DENV3, n = 12/22; DENV4, n = 3/5), and 1 had DwoWS with both DENV1 and DENV3 detected by RT-PCR. There were no differences in the demographics or baseline immune status between participants who developed DwoWS versus DWWS (Supplemental Table 4).

### Cross-reactive antibodies with symptomatic dengue

**Figure 1:**
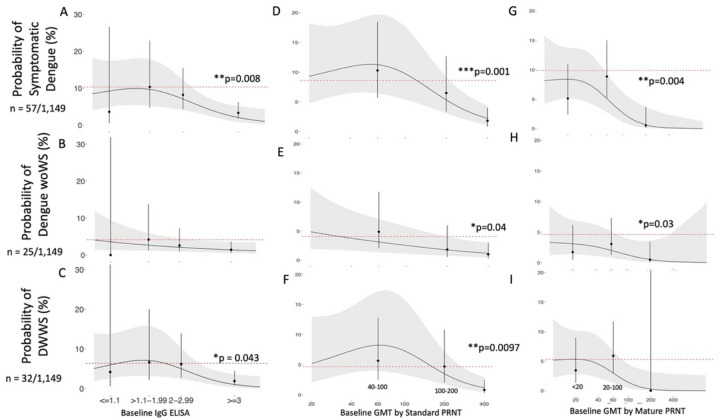
Probability of disease by baseline IgG ELISA (A-C) and baseline GMT measured by PRNT with standard reference (D-F) or mature clinical (G-I) strains. The probability of each disease outcome was modeled as a function of baseline antibody titer on both discrete and continuous scales. All continuous relationships were modeled using Poisson GAMs (continuous black line) with 95% CI (grey shading). Point estimates and CIs correspond to predicted probabilities from logistic regression models, and p-values correspond to antibody bins with a significantly different dengue risk compared to the naïve group. Dashed line indicates the disease probability in naïve individuals. DENV IgG ELISA was measured on all participants (n=1,206), and GMTs were measured on random subsets using standard reference (n = 823) and mature clinical strains (n=293). Inverse probability weighting was used to adjust for GMT subset size. All models were adjusted for age, sex, and enrollment site, and model estimates are shown for the average study participant (female, age 10, from Bogo).

We next evaluated whether the probability of experiencing dengue (DwoWS and DWWS) could be predicted by total binding antibodies measured at baseline using an IgG ELISA and neutralizing antibodies measured as the geometric mean of PRNT titers (GMT) against standard WHO DENV1–4 reference strains (standard) or mature clinical DENV1–4 isolates (mature). After adjusting for age, sex, and recruitment site, high total binding antibodies or nAb against standard or mature strains were associated with lower probabilities of dengue caused by any serotype as compared to the naïve group (Fig. 1). Specifically, the probability of dengue in the naïve group was 10% (95% CI: 5–22) versus 3% (2–6) among those with ELISA values ≥ 3 (p = 0.008). In the models examining PRNT GMTs, the probability of dengue was 9% (4–18) in the naïve group versus 2% (1–4) among those with a standard GMT > 200 (p = 0.001) and 1% (0–4) among those with mature GMT > 100 (p = 0.004). At lower antibody levels, none of the assays showed either enhancing or protective effects.

We also evaluated whether these same assays were associated with DwoWS or DWWS alone. Standard PRNT was associated with protection against both DWWS and DwoWS. IgG ELISA was associated with protection against DWWS, and the mature PRNT was associated with protection against DwoWS. Both assays also showed trends towards protection against DwoWS and DWWS, respectively. The association between dengue and high total binding antibodies and nAb suggests that these antibody measures are correlates of risk.

**Figure 2:**
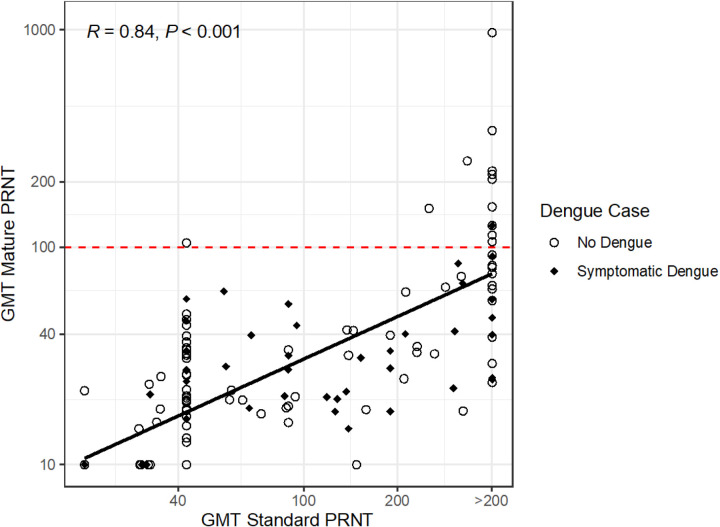
Correlation between GMTs as measured using PRNTs to standard reference and mature clinical strains. GMTs are log_10_ values plotted on a linear scale, and the correlation coefficient (R) was calculated using Pearson’s test with 95% CI. Only those with no dengue (open circles) or dengue (black diamond) and GMTs measured by both the standard and mature PRNTs are included (n = 256). Dashed red line indicates GMT=100 by mature assay.

To compare the antibodies measured by standard versus mature PRNT, we evaluated the association between the GMTs from these assays and found that they were highly correlated (R = 0.84). However, only 2% of cases occurred among those with mature GMT > 100, while 52% of cases occurred among those with standard GMT > 100 (p < 0.001) (Fig. 2). Notably, 24% of those with standard GMT > 200 had mature GMT > 100, and none of those with standard GMT < 200 had high mature PRNT. Thus, although correlated with the standard GMT, the mature GMT had a more consistent association with protection at the titers measured.

### Serotype-specific antibodies and risk of dengue

**Figure 3:**
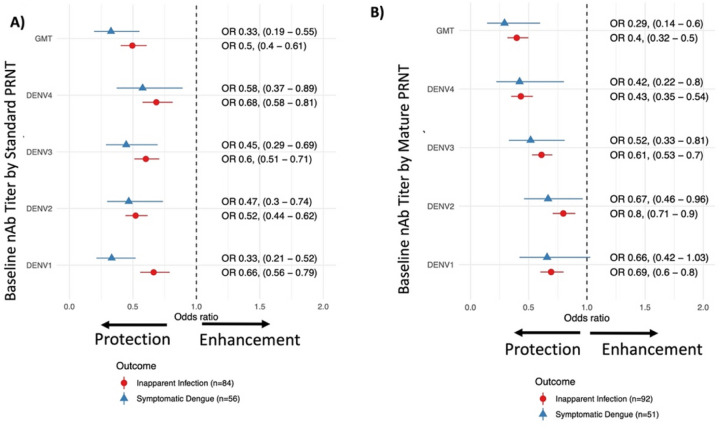
The odd ratios and 95% confidence intervals of inapparent infection (circles) and symptomatic dengue (triangle) by baseline GMT and serotype-specific nAbs measured with PRNT to standard reference (A) or mature clinical (B) strains. All models were adjusted for age, sex, and enrollment site, and inverse probability weighting was used to adjust for GMT and serotype-specific nAb subset sizes.

We next assessed both the GMT and titers to each serotype measured by standard and mature PRNT as predictors of symptomatic (over the 5-year follow up period) and inapparent (over the first 1–2 years of follow up) DENV infections caused by any serotype (Fig. 3). Both standard and mature GMTs were strongly protective with similar reductions in the odds of dengue per log_10_ increase in GMT (odds ratios ranged from 0.29–0.5 [CIs: 0.14–0.61]). Interestingly, titers to each of the four serotypes were also associated with decreased odds of inapparent and symptomatic infection caused by any serotype. The serotype-specific standard PRNT odds ratios ranged from 0.33–0.68 (0.29–0.89) and the mature PRNT odds ratios ranged from 0.42–0.8 (0.22–1.03) per unit increase in each log_10_ titer. Thus, both the GMT and titers to each serotype show protective effects when measured against dengue caused by any serotype, especially when measured by the standard PRNT.

**Figure 4:**
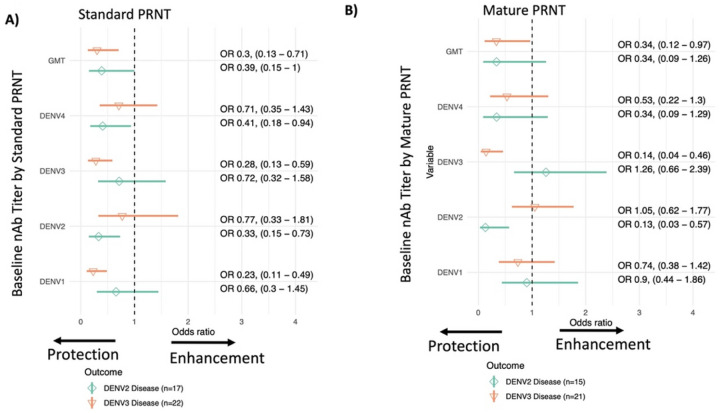
The odd ratios and 95% confidence intervals of dengue caused by DENV2 (diamond) and DENV3 (inverted triangle) by baseline GMT and serotype-specific nAbs measured with standard reference (A) or mature clinical (B) strains. All models were adjusted for age, sex, and enrollment site, and inverse probability weighting was used to adjust for GMT and nAb subset sizes.

To evaluate if the standard PRNT may identify more cross-reactive antibodies as compared to the mature PRNT, we next analyzed disease risk individually by serotype for cases caused by DENV2 and DENV3, the most common serotypes to cause disease in the cohort. Titers measured by the mature PRNT had strong protective effects against the matched serotype (Fig. 4). The odds of experiencing a DENV2 case was 0.13 (0.03–0.57) per log_10_ increase in mature DENV2 PRNT titer. Similarly, the odds of a DENV3 case were 0.14 (0.04–0.46) per log_10_ increase in mature DENV3 PRNT titer. The mature GMT did protect against DENV3, but only trended toward protection against DENV2, perhaps due to smaller number of DENV2 cases. For the standard PRNT, a similar pattern, although with a weaker effect size, was observed for DENV2 titers and reduced odds of DENV2 (odds ratio of 0.33 [0.15–0.73]), while titers to other serotypes and the GMT were not protective against DENV2. In contrast, while standard titers to DENV3 protected against DENV3 (odds ratio of 0.28 [0.13–0.59]), standard DENV1 titer and GMT were also associated with decreased odds of experiencing a DENV3 case. In sum, both standard and mature titers were highly protective against the matched serotype, but nAbs measured by mature PRNT appeared to have more serotype-specific protective effects.

We then measured the correlations among titers with the expectation that related (cross-reactive) antibody populations would have stronger associations than distinct (more type-specific) populations. The strongest correlation was observed between the standard DENV1 and DENV3 assays titers (r= 0.79, Supplemental Fig. 3A). In contrast, the other standard DENV titers had weaker correlations (r = 0.53–0.65), and the mature DENV titers had the weakest correlations among serotypes (r = 0.17–0.42, Supplemental Fig. 3B). Standard serotype-specific titers had stronger correlations with standard GMT (0.79–0.9) than mature serotype-specific titers had with mature GMT (0.58–0.77).

Previous studies have shown that DIII of the envelope protein and NS1 protein have serotype-specific and cross-reactive epitopes. Particularly, EDIII has major type-specific epitopes on the lateral ridge and subdominant cross-reactive epitopes. We thus evaluated whether magnitude of antibody binding to EDIII and NS1 from DENV2 and DENV3 predicted the risk of disease by the matched serotype. Neither EDIII nor NS1 binding antibodies were associated with risk (Supplemental Fig. 4).

### Antibody thresholds associated with protection against dengue

We assessed each antibody measure for a threshold associated with disease reduction (Table 2). While only the highest ELISA values (4.5) and standard GMT (> 200) were associated with a 70% disease reduction compared to the naïve group, a mature GMT of 84 was associated with 70% disease reduction. When serotype-specific protection was evaluated, titers measured by mature PRNT provided 70% protection at lower levels than the standard PRNT against DENV2 (40 vs. >200) or DENV3 (40 vs. 181). However, 90% reduction in disease was only observed at a mature GMT of 400, meaning some individuals still experienced disease with high mature PRNT titers.

### Impact of strain and maturation state on titers

**Figure 5:**
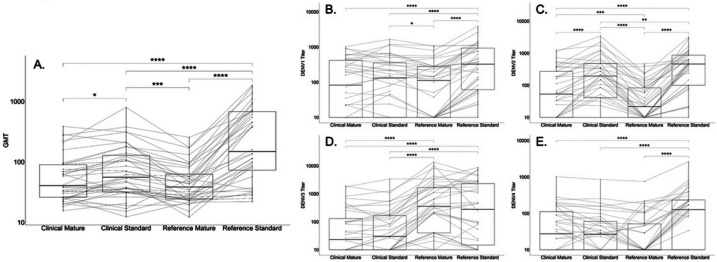
Geometric mean (A) and serotype specific (B-E) titers of each strain and maturation state combination. Horizontal lines connecting points track measurements from the same individual. Boxes indicate interquartile range and bold lines indicate median. N=36, *p<0.05, **p<0.01, ***p<0.001, ****p<0.0001; paired t-test with Bonferroni correction.

To assess the impact of viral strain as compared to maturation state on nAb titers, a direct comparison of titers measured against standard and mature reference and clinical strains was performed (Fig. 5). Across the four strain and maturation state combinations, the mean GMT was highest against the reference standard virus (195), followed by the clinical standard virus (67). The lowest GMTs were observed against the mature clinical (53) and mature reference strains (43), and these were similar to each other, suggesting that maturation state might be more important than strain to determining viral titers (Fig. 5A).

When stratified by serotype, the trends among strain and maturation states were more variable (Fig. 5B–E). Specifically, the reference standard viruses had the highest titers across serotypes, except against DENV3 where the titers measured by reference standard and reference mature viruses were similar (231 vs. 296, p = NS). In contrast, the reference mature strain induced the lowest titers for DENV2. The clinical standard and mature viruses induced similar titers for all serotypes, except DENV2, where the clinical mature virus induced the lower titers (DENV2 clinical standard: 141 vs. clinical mature: 60, p < 0.0001). Thus, maturation state may play a bigger role in determining titers for reference rather than clinical strains. Additionally, the clinical mature strains consistently yielded lower titers across serotypes and may be the most selective assay for measuring antibody neutralization.

## Discussion

We leveraged a large prospective, observational cohort to assess various antibody measures as potential correlates of risk for dengue. Total binding antibodies and both GMT and serotype-specific nAb measured with standard and mature viruses were associated with a decreased dengue risk. Standard and mature serotype-specific nAb titers had the strongest protective effects against the matched serotype, supporting the dogma that exposure to one DENV serotype induces excellent immunity to that type^32^. However, when compared to the standard serotype-specific titers, the mature titers had bigger effect sizes against matched serotypes and decreased cross-reactivity among titers, suggesting that these viruses bind more type-specific antibodies. Comparative PRNTs indicated that both maturation state and strain impact titer levels, but titers measured against the clinical mature strains had lower, more consistent thresholds associated with protection. Thus, in population and vaccine studies, the use of clinical and/or mature strains to measure titers may be preferable as correlates. In contrast, reference standard strains are more easily neutralized, potentially making them preferable for identifying any previous DENV exposure.

We found that high total IgG and GMT measured by standard and mature viruses decreased the risk of dengue. We did not observe any enhancing effects at low to medium total binding antibodies and GMT. This may be because our cohort had few cases overall and almost no severe dengue cases. Previous work demonstrating enhancement at lower titers evaluated larger numbers of cases and identified the strongest enhancement signal for severe disease manifestations^33^. GMT as measured with standard and mature viruses were also associated with reduced odds of inapparent infection, which could be important for examining viral outbreak potential. The protective effects observed with high total binding and nAbs are consistent with findings from inhibition ELISA and vaccine-specific PRNT assays^14, 34^.

The type of virus used in the assay seems to impact the accuracy of the correlate. Specifically, although GMT > 200 as measured using standard reference strains significantly decreased dengue risk, 38% of cases occurred among individuals with this antibody level. Additionally, 52% of cases occurred among those with a standard GMT > 100. This is aligned with previous work where standard vaccine strains were used to measure GMT, and GMT > 100 at month 13 post Dengvaxia was associated with 50% vaccine efficacy^14^. Conversely, antibodies as measured with the mature clinical strains had a lower threshold for protection and only 2% of cases occurred among those with GMT > 100. Notably, 78% of our cohort had multitypic immunity, and GMT is likely a more accurate correlate in individuals with this immune history. For monotypics, the GMT is the average of a positive titer against one serotype, and low titers against the other three. Thus, the GMT would be accurate except against the strain that induced monotypic immunity. In sum, GMT is likely most useful as a correlate when measured with mature clinical strains in more multitypic populations.

Among all the antibodies and cases assessed, the mature clinical serotype-specific nAb titer against the matched serotype had the strongest effect sizes, and correlation analyses suggest that mature clinical virions bind more type-specific antibodies than reference standard strains. The strong protective effects of matched serotype-specific nAb are consistent with previous reports that type-specific antibodies correlate with protection^21^. Additionally, the highest effect sizes with the mature clinical strains suggest that these virions bind more potent antibodies. Interestingly, heterotypic titers were not associated with protection against DENV2 or DENV3 except that standard titers against DENV1 did decrease DENV3 disease risk. This protective effect and the tight correlation between the standard DENV1 and DENV3 titers suggest that these virions are likely binding some of the same antibodies. Moreover, all serotype-specific standard nAb were strongly correlated with GMT (r ≥ 0.79), while mature serotype-specific nAb had weaker correlations with GMT (r < 0.79). Tight associations between serotype-specific nAb and GMT are likely due to standard virions binding cross-reactive antibodies. In contrast, weaker correlations among clinical mature serotype-specific titers likely reflect more selective antibody binding. We hypothesize that some of this discrimination is due to the structure of mature virions with their less accessible fusion loop, but the use of clinical strains may also contribute. We also evaluated whether the magnitude of antibodies binding to certain antigens that are thought to be the targets of type-specific protection, including EDIII and NS1, were associated with dengue disease and found they were not strong predictors. This finding suggests that measuring a broader antibody pool may be important for dengue immune correlates.

Neutralization assays assessing the impact of strain and maturation state on titers revealed that the reference standard viruses had the highest titers, except against DENV3, where they were similar to the reference mature titers. Conversely, the reference mature virus induced the lowest titers against DENV2. Clinical standard and mature viruses induced similar titer levels, except against DENV2, where the clinical mature titers were lower. High titers against reference standard strains are consistent with previous work indicating that highly laboratory passaged DENVs are the most sensitive to neutralization^21^. The significant differences in titers measured among all DENV2 strains may reflect increased differences in epitope exposure and sensitivity to neutralization due to viral breathing^35^. DENV2 aside, the consistent titers among clinical strains suggests that these may bind primarily higher quality antibodies in either maturation state. Thus, maturation state may have less influence on titer and virus neutralization when non-DENV2 clinical strains are used. Although these conclusions require confirmation with larger datasets examining more serotypes, this work suggests that clinical and mature strains are less sensitive to neutralization.

The decreased sensitivity to neutralization may result in the identification of higher quality antibodies measured by mature and clinical strains, thereby lowering the threshold for detecting disease reduction. Specifically, while an ELISA value of 4.5 and a standard GMT > 200 were associated with 70% disease reduction, mature GMT of 84 provided that level of protection. Additionally, only titers measured with the mature clinical strains were associated with 90% disease reduction. However, reaching 90% disease reduction required very high titers suggesting that titers measured with the mature clinical strains may still be an unsatisfying correlate. Moreover, these are time intensive assays requiring specialized labor, which also may limit their utility for large studies.

Our study has several limitations. Given the difficulties of performing PRNTs, our data are limited by the use of random subsets and two-dilution rather than full dilution assays for the standard reference strain. The two-dilution PRNT allowed us to estimate titers on more individuals, has been validated in a separate cohort^36^, and we found strong correlations between full titers and estimated two dilution titers when assessed with the mature clinical data (Supplemental Fig. 1). Inverse probability weighting was used to complete the datasets, and this was validated by comparing the estimates obtained using this method versus those from bootstrapping and from the full dataset using the ELISA measures (Supplemental Fig. 2). Additionally, our cohort included no severe dengue cases, a high percentage of individuals with multitypic immunity, and infections mostly with DENV2 and DENV3. Thus, nAb thresholds associated with protection may differ in other populations and with the virus strain chosen for the assay. Despite their limitations, these data provide compelling support for the use of mature and clinical isolates in PRNTs and for titers obtained from these assays to serve as correlates of risk.

Overall, an accurate and reproducible correlate of protection would be a valuable contribution to the development of dengue vaccines. After adjusting for age, sex, and enrollment location, we demonstrate that ELISA IgG, GMT, and serotype-specific nAb titers measured by the standard and mature assays serve as correlates of risk for inapparent and symptomatic DENV infection. Mature and clinical strains likely identify higher quality nAbs given the virion structure and similarities with infecting strains as evidenced by a lower, more consistent threshold associated with protection. Thus, nAb titers measured with mature and clinical isolates may be a helpful though exacting correlate of protection, and this should be further validated in other studies.
